# Which risk factors determine cartilage thickness and composition change in radiographically normal knees? – Data from the Osteoarthritis Initiative

**DOI:** 10.1016/j.ocarto.2023.100365

**Published:** 2023-04-28

**Authors:** F. Eckstein, S. Maschek, A. Culvenor, L. Sharma, F.W. Roemer, G.N. Duda, W. Wirth

**Affiliations:** a Department of Imaging and Functional Musculoskeletal Research, Institute of Anatomy and Cell Biology & Ludwig Boltzmann Intitute of Arthritis & Rehabilitation (LBIAR), Paracelsus Medical University Salzburg & Nuremberg, Salzburg, Austria; b Chondrometrics GmbH, Ainring, Germany; c La Trobe Sport and Exercise Medicine Research Centre, School of Allied Health La Trobe University, Bundoora, Australia; d Department of Medicine, Northwestern University Feinberg School of Medicine, Chicago IL, USA; e Department of Radiology, Friedrich-Alexander University Erlangen-Nürnberg & Universitätsklinikum Erlangen, Erlangen, Germany; f Department of Radiology, Boston University School of Medicine, Boston, MA, USA; g Julius Wolff Institute, Berlin-Brandenburg Institute of Health at Charité – Universitätsmedizin Berlin, Germany

**Keywords:** Cartilage, Cartilage thickness, Cartilage loss, Transverse relaxation time, Magnetic resonance imaging (MRI), Risk factors

## Abstract

**Objective:**

Therapy for osteoarthritis ideally aims at preserving structure before radiographic change occurs. This study tests: a) whether longitudinal deterioration in cartilage thickness and composition (transverse relaxation-time T2) are greater in radiographically normal knees “at risk” of incident osteoarthritis than in those without risk factors; and b) which risk factors may be associated with these deteriorations.

**Design:**

755 knees from the Osteoarthritis Initiative were studied; all were bilaterally Kellgren Lawrence grade [KLG] 0 initially, and had magnetic resonance images available at 12- and 48-month follow-up. 678 knees were “at risk”, whereas 77 were not (i.e., non-exposed reference). Cartilage thickness and composition change was determined in 16 femorotibial subregions, with deep and superficial T2 being analyzed in a subset (n ​= ​59/52). Subregion values were used to compute location-independent change scores.

**Results:**

In KLG0 knees “at risk”, the femorotibial cartilage thinning score (−634 ​± ​516 ​μm) over 3 years exceeded the thickening score by approximately 20%, and was 27% greater (p ​< ​0.01; Cohen D −0.27) than the thinning score in “non-exposed” knees (−501 ​± ​319 ​μm). Superficial and deep cartilage T2 change, however, did not differ significantly between both groups (p ​≥ ​0.38). Age, sex, body mass index, knee trauma/surgery history, family history of joint replacement, presence of Heberden's nodes, repetitive knee bending were not significantly associated with cartilage thinning (r^2^<1%), with only knee pain reaching statistical significance.

**Conclusions:**

Knees “at risk” of incident knee OA displayed greater cartilage thinning scores than those “non-exposed”. Except for knee pain, the greater cartilage loss was not significantly associated with demographic or clinical risk factors.

## 1 Introduction

Therapy for osteoarthritis (OA) ideally aims at preserving synovial joint structure before radiographic change (ROA) occurs. In this context, articular cartilage represents a primary treatment target, as it ensures proper joint function by providing an almost frictionless gliding surface that, by virtue of hydrostatic pressurization [1], distributes mechanical loads very evenly from one limb segment to the other. In order to fulfill this function, a certain tissue thickness, as well as specific compositional and mechanical properties need to be met.

It is assumed that loss in cartilage thickness, and longitudinal change in cartilage composition, commence before signs of ROA become apparent. As it would be unreasonable, and potentially harmful, to treat everyone with radiographically normal knees preventively, it is important to validate risk factors that put people at risk of cartilage loss and/or composition change, in order to apply targeted preventive therapy that slows down or even stops OA development or progression. MRI spin–spin or transverse relaxation time (T2) has been used as a marker of cartilage composition, and has specifically been proposed to detect structural cartilage pathology before the onset of ROA and cartilage matrix loss [[2], [3], [4]]. Cartilage T2 has been suggested to reflect collagen integrity, orientation, and hydration [2,4,5] and has been shown to be associated with cartilage histological grading [6,7] and mechanical properties [2,8]. Based on the assumption that risk factors or incident knee OA also represent risk factors of longitudinal cartilage thickness loss and cartilage composition (T2) change, both measures were compared between knees “at risk” and those “non-exposed” to such risk factors of incident knee OA. Additionally, radiographic joint space width (JSW) was included, as it has served as a reference method of OA structural progression.

There are methodological challenges in studying the effect of risk factors of knee OA progression, once incident ROA has occurred [9]. Observational studies have identified multiple risk factors associated with an increased risk for incident knee ROA; however, whether these risk factors also are important in ROA progression has remained partly unclear. Evaluation of the effect of risk factors of ROA progression in knees with established disease is typically performed by comparing knees with the risk factor to control knees without that risk factor. However, in these controls, incident ROA has obviously occurred despite the lack of the known risk factors that are assumed to also drive progression. Yet, other unidentified risk factors, for instance genetic factors, may have led to incident ROA in these controls, and these risk factors may also favor progression and thus act as confounders. This so-called “collider bias” renders it challenging to statistically identify the effect of a risk factor of OA progression, once incidence of ROA has occurred [9]. Therefore, studying the effect of risk factors on a quantitative measure of structural progression that is sensitive to change both before and after incident ROA, and that regards “progression” as a continuum of both stages may provide a unique opportunity to circumvent these methodological challenges in identifying risk factors of progression.

The current study was designed to test a) whether longitudinal change in cartilage thickness or composition (and that of radiographic JSW) is greater in radiographically normal knees “at risk” of incident ROA than in those without such risk factors, and b) which specific risk factors may be associated with such a difference. Given relatively small rates of cartilage change over time in KLG0 knees, a relatively long (3 year) longitudinal observation period was chosen.

## 2 Methods

### 2.1 Participant selection

The current study was based on data from the Osteoarthritis Initiative (OAI), a prospective, observational cohort study (http://www.oai.ucsf.edu/, clinicaltrials.gov identifier: NCT00080171). The OAI enrolled 4796 participants aged 45–79 years, in order to collect clinical data, 3 ​T magnetic resonance images (MRIs) and fixed-flexion radiographs from both knees at four clinical sites [10]. The OAI was approved by the Committee on Human Research, the Institutional Review Board (IRB) for the University of California, San Francisco (UCSF) and the IRBs at each clinical site. Radiographic selection of the sample studied here relied on the central radiographic readings performed at Boston University (version 0.7/1.7) [11].

The current study encompassed 755 subjects who were all bilaterally radiographically normal (i.e. Kellgren Lawrence grade [KLG] 0) at 12-month follow-up (the beginning of the observation period), and had MRIs available at 12- and 48-month follow-up. 678 of these subjects were from the so-called *incidence cohort* of the OAI (cohort assignment v 25) and were “at risk” of incident knee OA by factors such as obesity, previous knee trauma (defined as having injured the knee so badly that it was difficult to walk for at least one week), previous knee surgery, family history of total knee joint replacement (TKR), presence of Heberden's nodes (at least grade 3), engagement in at least one frequent repetitive knee activity, or knee pain (below called “at risk” cohort). The other 77 subjects were from the so-called “non-exposed” healthy reference cohort (below called “non-exposed” cohort) who were bilaterally KLG0 and in whom the above risk factors were excluded [12]. Further, these “non-exposed” subjects were aged 45–79 years, and had no pain, aching, or stiffness in either of both knees [12].

**Fig. 1:**
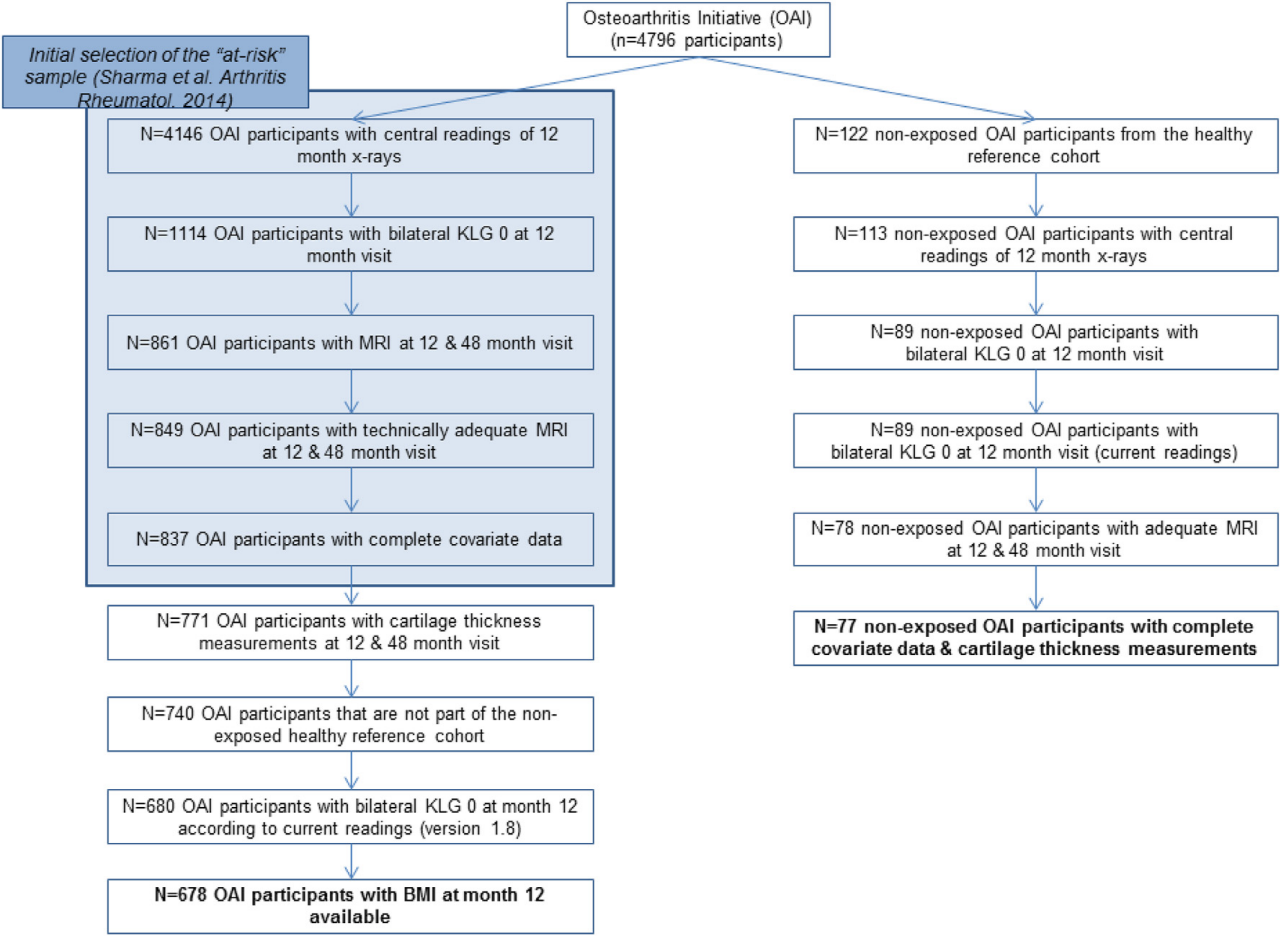
Flow chart visualizing the participant selection process from the Osteoarthritis Initiative (OAI) for the current study.

The current study did not rely on the initial selection of “non-exposed” participants made by the sites (n ​= ​122), but on those in whom bilateral KLG0 status was confirmed by central radiographic readings from a team of three expert radiologists/rheumatologists at Boston University. Of 112 of the initial “non-exposed” subjects with at least one follow-up MRI [13], 89 had both knee radiographs centrally assessed as KLG0 at both baseline and 12 month (M) follow-up (the baseline time point of the present observation interval [11]). Of these, 77 had MRIs available at both 12- and 48-month follow-up. The 678 representatives of the “at risk” cohort studied here represented a random selection of a total of 746 OAI participants from the OAI *incidence cohort* who were bilaterally KLG0 at baseline and Y1 follow up (central readings) and had 12- and 48-month follow-up MRI data available for longitudinal analysis. The selection was not based on specific criteria, but by omitting cases at the end of the full reading list due to budget restrictions [11]. The selection process is visualized in a flow chart (Fig. 1). Knee pain frequency classification was based on the right and left knee symptom status [10], with knees categorized: a) pain, aching or stiffness on most days of at least one month in the past 12 months (frequent pain); b) pain, aching or stiffness in the past 12 months, but not on most days of a month (infrequent pain); c) no pain, aching or stiffness in the past 12 months. Further, the numerical rating scale (NRS) pain level (0–10) for the past month, and the Western Ontario and McMaster Universities Osteoarthritis Index (WOMAC) pain scores for the past 7 days were available for both knees and used for the current analysis. Clinical data of the 12-month follow-up relied version 1.2.2, and those of 48-month follow-up version 6.2.1.

### 2.2 Cartilage thickness and radiographic joint space width (JSW) measurements

Femorotibial cartilage thickness measurement in one knee of each participant (the right knee, if MRI was available, otherwise the left knee) was based on segmentation of sagittal DESS MRIs, and computation using Chondrometrics software (Chondrometrics GmbH, Ainring, Germany), as described previously [14]. The mean cartilage thickness (ThCtAB.Me) was determined for the total femorotibial joint (FTJ) as well as the medial and lateral compartment (MFTC/LFTC) and in 16 femorotibial subregions: 10 tibial (central, external, internal, anterior, posterior in the medial and lateral tibia, respectively), and 6 femoral (central, external, internal in the medial and lateral, weight-bearing femur, respectively) [15]. Location-independent cartilage thinning and thickening scores were computed by summing all negative/positive changes across the 16 subregions within each knee [16]. Because of their greater statistical efficiency [17], ordered values (OVs) were computed by ordering the subregional changes in each knee in ascending order, with OV1 representing the subregion with the largest thickness loss and OV16 that with the largest thickness gain.

Radiographic JSW was measured quantitatively from weight-bearing fixed flexion radiographs in those in which radiographic measures were available, as described previously [10]. Specifically, the minimum of the JSW was determined in the medial femorotibial compartment, as well as the JSW at a fixed location in the medial (x ​= ​0.225 [22.5% of the distance from the medial to lateral femoral margin]) and in the lateral compartment (x ​= ​0.750 [75.0% of the above distance] [10]) JSW data of the 12-month follow-up relied version 1.8, and those of 48-month follow-up version 6.5.

### 2.3 Cartilage transverse relaxation time measurement (T2)

Femorotibial cartilage T2 measurement was based on segmentation of sagittal MESE MRIs [10,18] acquired in right knees at 12- and 48-months, and computation using in-house software [19,20]. This analysis was performed in a subset of 59 of the 678 “at risk” knees analyzed for a previous study on cartilage T2 change [21], excluding one knee in that study that did not have measures of cartilage thickness change, and in all knees of the “non-exposed” reference cohort that were bilaterally KLG0 (central readings) at both baseline and 12-month follow-up and that had complete demographic data and usable MESE acquisitions available at 12- and 48-month follow-up (n ​= ​52). The demographics, JSW and cartilage characteristics for this subcohort are described in Suppl. Table 1. Because T2 is known to vary with cartilage depth [2,22], the top (superficial) and bottom (deep) 50% of the cartilage were analyzed separately [20]. T2 was computed for each voxel by fitting a mono-exponential decay curve to the measured signal intensities, with the 10 ​ms echo being excluded to reduce impact of stimulated echoes [2,20]. Deep and superficial T2 was also determined across the same regions described above, with location-independent T2 lengthening and shortening scores and OVs being derived in the same way as for cartilage thickness [16,23]. OV16 represented the subregion with the greatest T2 increase, with T2 lengthening indicating matrix deterioration.

### 2.4 Statistical analysis

Given that, in KLG0 knees, no compartment or location can be assumed to be particularly “vulnerable”, the primary analytic focus of this study was the comparison of the location-independent cartilage “thinning score” between the 678 “at risk” knees and the 77 “non-exposed” knees; the co-primary focus was the cartilage “thickening score”. The secondary analytic focus was the comparison of the T2 “lengthening score”, and the co-secondary focus the T2 “shortening score”. Between-group comparisons were performed using independent sample t-tests. Comparisons for all other measures of cartilage thickness and composition change (compartments, plates, subregions, and OVs) were considered exploratory. As outlined above, the T2 analysis was performed in a subsample, and a sensitivity analysis of cartilage thickness measures was performed in the same (smaller) sample so that the findings could be compared directly.

Then, differences in age, sex, body mass index (BMI), NRS and WOMAC knee pain between the “non-exposed” and the “at risk” groups were tested for statistical significance using independent sample t-tests, non-parametric Mann–Whitney U tests and chi [2] tests as appropriate, and effect sizes described by Cohen's D. In a next step, the relationship of the risk factors with the variability of the cartilage thinning score in the “at risk” cohort was studied using univariate linear regression models, and the same was done for the “at risk” and “non-exposed” cohorts combined. A multivariate model with all risk factors included was analyzed to determine how much of the cartilage thinning variability was explained by all risk factors. Regression models were analyzed to ensure that general assumptions, including linearity, homoscedasticity and normality of residuals were met. Three outliers with standardized residuals >±2.5 were excluded from regression analyses.

## 3 Results

### 3.1 Baseline demographic, radiographic, and cartilage status

In the 678 “at risk” participants, 677 right and 1 left knee were studied; 384 (57%) were from women and 294 from men (90.1% white/Caucasian, 8.0% black/African American, 1.9% other). In the “non-exposed” reference cohort, 77 right knees were analyzed; 46 were from women (60%) and 31 from men. The ratio between men and women did not differ between both groups (p ​= ​0.60). In the “at risk” cohort, 171 (25%) reported a relevant past injury of the knee studied, 37 (5.5%) previous surgery of the knee studied, 113 (17%) a family history TKR replacement, 169 (25%) presence of Heberden's nodes, and 493 (73%) frequent repetitive knee bending activity; in 139 (21%) the knee studied was frequently painful, 302 (45%) infrequently painful, and 234 (34%) did not have knee pain at 12-month follow-up. Table 1 lists baseline demographic, radiographic and MRI cartilage variables for both cohorts, and Suppl. Table 1 those for the subsample in which T2 was analyzed, including T2 results at the beginning of the longitudinal observation period (12-month follow-up).

**Table 1:** Baseline data in Kellgren Lawrence grade (KLG) 0 knees “at risk” of incident knee OA vs. “non-exposed” KLG0 reference knees.

	“At risk” (n ​= ​678)					“Non-exposed” (n ​= ​77)					Difference	
	Mean	SD	95% CI		N	Mean	SD	95% CI		N	p	Cohen D
Age (years)	59.6	8.8	58.9	60.3	678	55.4	7.3	53.7	57.0	77	<0.001	0.49
BMI (kg/m^2^)	26.7	4.2	26.4	27.0	678	24.4	3.2	23.6	25.1	77	<0.001	0.56
NRS (0–10)	1.7	2.1	1.5	1.9	673	0.2	0.5	0.0	0.3	77	<0.001	0.76
WOMAC pain (0–20)	1.2	1.9	1.0	1.3	678	0.1	0.4	0.0	0.2	77	<0.001	0.58
ThC FTJ (mm)	7.3	1.0	7.3	7.4	678	7.2	1.1	7.0	7.5	77	0.407	0.10
ThC MFTC (mm)	3.4	0.5	3.4	3.5	678	3.4	0.5	3.3	3.5	77	0.263	0.13
ThC LFTC (mm)	3.9	0.6	3.8	3.9	678	3.8	0.6	3.7	4.0	77	0.626	0.06
Min JSW (mm)	4.6	0.7	4.5	4.8	146	4.7	0.8	4.5	4.9	77	0.582	−0.08
Medial FL JSW (mm)	5.7	0.9	5.6	5.9	146	5.6	0.8	5.4	5.8	77	0.310	0.14
Lateral FL JSW (mm)	7.5	1.3	7.3	7.7	198	7.5	1.3	7.2	7.8	73	0.975	0.00

BMI ​= ​body mass index; NRS ​= ​numerical rating scale (0–10, with 0 being no pain, and 10 the worst pain), WOMAC ​= ​Western Ontario & Mc Master Universities Osteoarthritis Index pain component (0–20, with 0 being no pain, and 20 the worst pain); ThC ​= ​thickness of cartilage; FTJ ​= ​total femorotibial joint (ThC FTJ ​= ​ThC MFTC ​+ ​ThC LFTC); MFTC ​= ​medial femorotibial compartment (ThC MFTC ​= ​ThC medial tibial ​+ ​ThC medial weight-bearing femur; LFTC ​= ​ThC lateral tibial ​+ ​ThC lateral weight-bearing femur; min JSW ​= ​minimum radiographic joint space width determined from fixed flexion radiographs; FL ​= ​fixed location: medial, X ​= ​0.225; lateral, X ​= ​0.750.

Differences in age, BMI, NRS pain, and WOMAC pain between the “at risk” and the non-exposed cohort were statistically significant (p ​< ​0.001; Table 1), whereas no significant differences in baseline radiographic and cartilage thickness parameters were found (Table 1).

Of the 678 knees of the “at risk” cohort, 11 were classified as KLG1 at the end of the observation period (48-month follow-up), 5 KLG2, and 4 KLG3 or 4, whereas 621 (92%) remained KLG0; 37 did not have a radiographic classification at this time point. Of the 77 knees of the “non-exposed” cohort, 3 were classified as KLG1 at 48-month follow-up, 2 KLG2, and none KLG3 or 4, whereas 68 (88%) remained KLG0; 4 had no classification. At the last time point at which knee were radiographically evaluated in the OAI (96-month follow-up), 49 were classified as KLG1 15 KLG2, and 13 KLG3 or 4 in the “at risk” cohort, whereas 476 (70%) remained KLG0; 125 did not have a radiographic classification at this time point. In the “non-exposed” cohort, 4 were classified as KLG 1 at 96-month follow-up, 2 KLG2, and none KLG3 or 4, whereas 68 (75%) remained KLG0; 13 had no classification.

### 3.2 Longitudinal change in cartilage thickness and radiographic measures

**Fig. 2:**
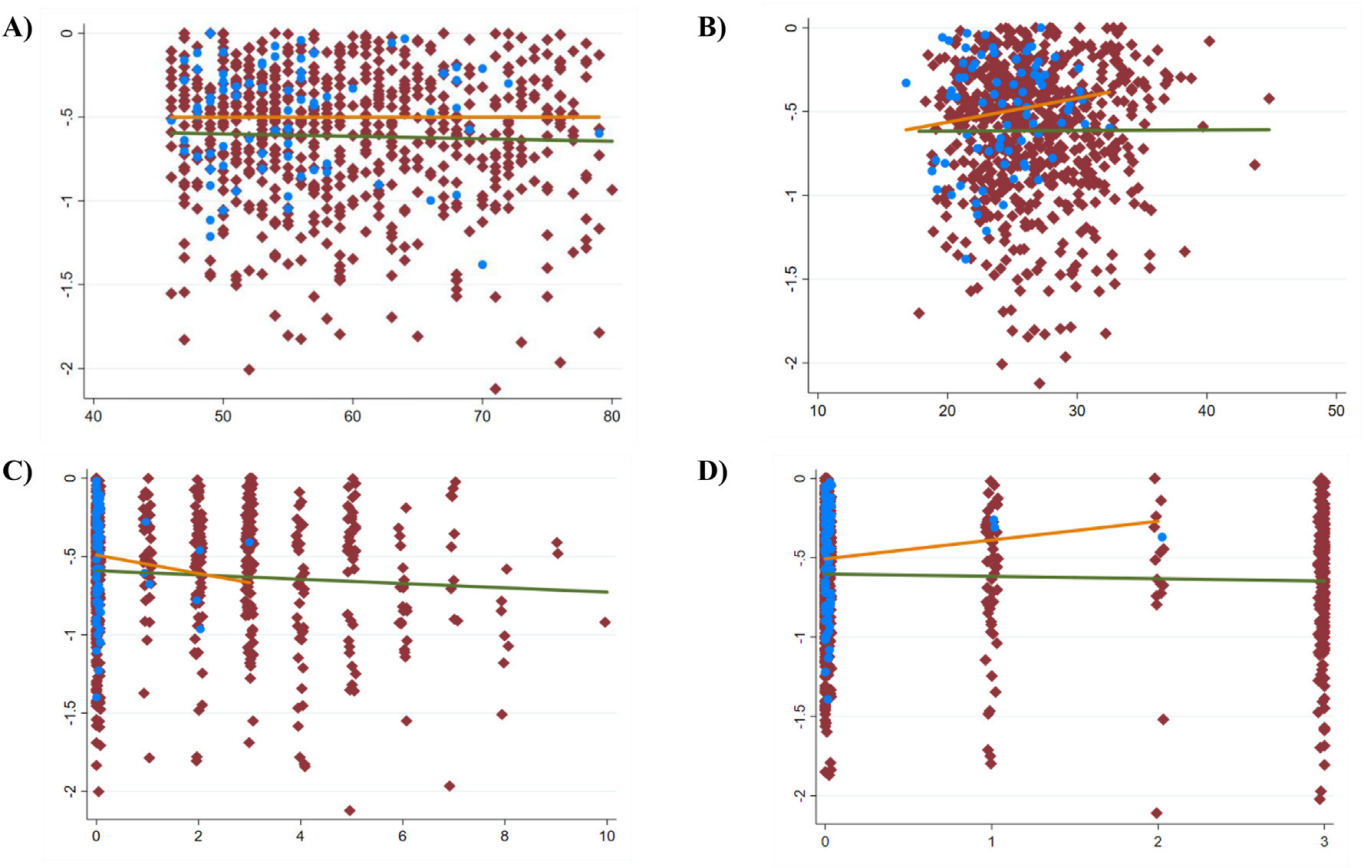
Cartilage thinning scores in mm (y-axis) in the “at risk” (red symbols, orange line ​= ​fitted values) and in the “non-exposed” cohort (blue symbols, green line ​= ​fitted values) as related to A) Age; B) BMI; C) NRS pain, and D) Heberden's nodes; three outliers in the thinning scores have been removed from the display for better visualization.

**Fig. 3:**
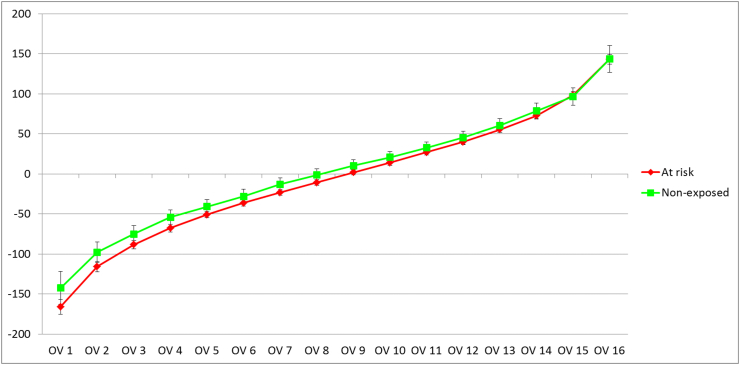
Cartilage thickness change (in μm) over an observation period of 3 years across 16 ordered values (OVs) of femorotibial subregion. OV 1 ​= ​subregion with the greatest cartilage loss in each knee; OV16 ​= ​subregion with the least cartilage loss or most cartilage thickening in each knee. The cohort “at risk” of incident knee OA is shown in red, the “non-exposed” reference cohort without risk factors in green. The results for subregion averages are provided in Suppl. Table 1.

The MRI-derived location-independent cartilage thinning score (ThC_TnS) was statistically significantly greater than that in “non-exposed” cohort (p ​= ​0.027; Cohen D ​= ​0.27), whereas the thickening score (ThC_TkS) was similar between the two (Table 2). In the “at risk” cohort, the magnitude of the thinning score exceeded the thickening score by approximately 20%, whereas in the “non-exposed” cohort it was 7% less than that of the thickening score; amongst those with a thinning score of −1.5 ​mm or greater there only were subjects from the “at risk” cohort and none from the “non-exposed” cohort (Fig. 2). Nine (of the 16) OVs suggested greater thinning or less thickening in the “at risk” cohort than in the non-exposed cohort (Fig. 3), whereas only two subregions (cLT; icMF) suggested greater thinning, and two subregions (iMT, ccMF) less thickening (p ​< ​0.05, not adjusted for multiple comparison; Suppl. Table 2). Also, change in the total, medial or lateral femorotibial joint cartilage thickness did not differ significantly between the “at risk” and the “non-exposed” cohort (p ​≥ ​0.15; Cohen's D ​≤ ​0.17; Table 2). The change in minimum and (medial and lateral) fixed location joint space width was significantly different from zero in both cohorts, but also did not differ significantly between the two (Table 2).

**Table 2:** Three-year change in radiographic measures, cartilage thickness and composition (T2) in Kellgren Lawrence grade (KLG) 0 knees “at risk” of incident knee OA vs. “non-exposed” KLG0 reference knees.

	“At risk”					“Non-exposed”					Difference	
	Mean	SD	95% CI		N	Mean	SD	95% CI		N	p	Cohen D
ThC thinning Score (μm)	−634	516	−673	−596	678	−501	319	−573	−429	77	0.03	−0.27
ThC thickening Score (μm)	529	366	501	556	678	537	308	467	607	77	0.85	−0.02
ThC FTJ (μm)	−15	186	−29	−1	678	17	138	−15	48	77	0.151	−0.17
ThC MFTC (μm)	2	112	−6	11	678	18	77	1	36	77	0.230	−0.14
ThC LFTC (μm)	−17	117	−26	−8	678	−1	99	−24	21	77	0.257	−0.14
Min JSW (μm)	−229	633	−333	−124	144	−201	403	−295	−107	73	0.73	−0.05
Medial FL JSW (μm)	−290	608	−390	−190	144	−266	419	−363	−168	73	0.76	−0.04
Lateral FL JSW (μm)	−285	692	−383	−187	194	−222	537	−351	−93	69	0.49	−0.10
T2 deep lengthening score (ms)	20.7	12.1	17.5	23.8	59	19.7	13.9	15.8	23.6	52	0.70	0.07
T2 deep shortening score (ms)	−17.8	16.3	−22.1	−13.6	59	−15.5	12.8	−19.0	−11.9	52	0.40	−0.16
T2 Sf lengthening score (ms)	27.1	16.8	22.8	31.5	59	24.4	15.7	20.0	28.8	52	0.38	0.17
T2 Sf shortening score (ms)	−14.7	9.3	−17.1	−12.2	59	−13.8	10.7	−16.8	−10.8	52	0.65	−0.09

ThC ​= ​thickness of cartilage; FTJ ​= ​total femorotibial joint (ThC FTJ ​= ​ThC MFTC ​+ ​ThC LFTC); MFTC ​= ​medial femorotibial compartment (ThC MFTC ​= ​ThC medial tibial ​+ ​ThC medial weight-bearing femur; LFTC ​= ​ThC lateral tibial ​+ ​ThC lateral weight-bearing femur; min JSW ​= ​minimum radiographic joint space width determined from fixed flexion radiographs; FL ​= ​fixed location: medial, X ​= ​0.225; lateral, X ​= ​0.750; T2 ​= ​MRI transverse (spin–spin) relaxation time; deep ​= ​deep cartilage layer (50%), sf ​= ​superficial cartilage layer (50%).

### 3.3 Longitudinal change in cartilage composition (T2)

In the subset of the “at risk” cohort (n ​= ​59), the deep and superficial cartilage T2 lengthening scores (dT2_LeS) exceeded the shortening scores in the same layer, but the same was observed in the “non-exposed” cohort (n ​= ​52; Table 2). There was no statistically significant difference in the lengthening or shortening scores in each of the two layers (p ​≥ ​0.37). Also, studying T2 changes in the superficial and deep layer in the total, medial or lateral femorotibial joint did not reveal any statistically significant differences between both cohorts (p ​≥ ​0.25; Cohen's D ​≤ ​0.28). Yet, in the above subset of both cohorts (n ​= ​59 vs. 52) the cartilage thinning score differed significantly (−689 ​± ​438 ​μm [95% CI −803, −574] vs. −470 ​± ​295 ​μm [−552, −388); p ​= ​0.003; Cohen's D ​= ​0.59).

### 3.4 Risk factor associations with longitudinal cartilage change

There was no statistically significant association between the single risk factor and the cartilage thinning score in the “at risk” cohort, with all beta values falling below ±0.04 and r^2^ values below 1% (age: p ​= ​0.41; BMI: p ​= ​0.93; sex: p ​= ​0.73; previous knee injury: p ​= ​0.45; previous knee surgery: p ​= ​0.78 [p ​= ​0.52 for both combined]; family history of TKR: p ​= ​0.59; presence of Heberden's nodes: p ​= ​0.21, frequent knee bending activity: p ​= ​0.68; pain frequency: p ​= ​0.31; WOMAC pain: p ​= ​0.49), and only NRS pain reached borderline statistical significance (p ​= ​0.06). When including both the “non-exposed” and the “at risk” cohort in the analysis, the results were similar, with NRS pain becoming statistically significant (p ​= ​0.01; Fig. 2). A model consisting of age, sex, BMI, previous injury and/or surgery, family history of TKR, presence of Heberden's nodes, frequent knee bending activity, and pain frequency only explained <1% of the variability in the cartilage thinning score (r^2^) in the “at risk” cohort, and in the “non-exposed cohort” and “at risk” cohort taken together.

## 4 Discussion

This study explored, for the first time, whether clinical risk factors are associated with cartilage thickness and composition (T2) change in radiographically normal (KLG0) knees, prior to the onset of ROA. The location-independent cartilage thinning score was found to be significantly greater in a cohort “at risk” of incident ROA than in “non-exposed” reference cohort that did not express these risk factors. However, this difference could not be statistically related to any of the specific demographic or clinical risk factors, based on which the selection of the “at risk” cohort was made, except for NRS pain. In contrast to the cartilage thinning score, no differences in the longitudinal change of compartment-specific cartilage thickness, radiographic JSW, or superficial or deep cartilage T2 were noted between the “at risk” and the “non-exposed” reference cohort.

A limitation of the current analysis is some restriction with regard to generalizability: The OAI is not a community-based study that is representative of the general population, and there is some likelihood of ascertainment bias, in particular with regard to the “non-exposed” reference group that may actually represent “super-controls” given the general absence not only of radiographic OA and pain, but also of important risk factors of incident knee OA. On the other hand, these “super-controls” provide a unique opportunity to study the impact of general clinical risk factors before the actual onset of disease. Another limitation is that, due to budget restrictions, only 91% of the knees of the “at risk” cohort were studied. However, this was by a random, not systematic, criterion, and the main findings of the study (differentiation of “at risk” and “non-exposed” knees by the thinning score) were reproduced in the much smaller subcohort in which T2 measurements were available; therefore it is highly unlikely that this finding was impacted by selection bias.

A specific limitation of the T2 analysis component of the study is that only a subset of the “at risk” cohort was studied. However, the difference in cartilage thinning observed in the full cohort was also observed in the smaller subset of knees for which cartilage T2-measurements were available, so that the results appear to be robust, and the lack of detecting a difference in longitudinal T2 change is unlikely to be attributed to selection bias or a smaller statistical power. The same location-independent analysis methodology was used here to report lengthening of cartilage T2 [21], which is thought to be associated with compositional and mechanical deterioration of the cartilage [2,[4], [5], [6], [7], [8]]. Yet, in contrast to the cartilage thinning score, we were unable to detect significant differences in longitudinal change of cartilage T2 between KLG0 knees at risk and those not at risk of incident knee OA, both in the deep and in the superficial cartilage layer. This also was the case for measures of the total, medial and lateral femorotibial compartment. This is in some contrast to the assumption that cartilage T2 can detect structural cartilage pathology well before the onset of cartilage matrix loss [[2], [3], [4]]. This may be potentially associated with the lower precision (reproducibility) of T2 relaxometry in relation to cartilage thickness analysis [24], which may render it difficult to detect small differences in T2 change between different risk cohorts longitudinally. Another potential reason for the T2 changes not to significantly differ between both groups is that longitudinal changes in hydration or collagen (to which T2 should be sensitive) are too small, or may cancel each other out (i.e. changing the T2 in opposite directions) for revealing relevant longitudinal differences. Yet, in a previous study of KLG0 knees with contralateral radiographic joint space narrowing (JSN) [21], the deep layer T2 lengthening score as well as T2 in the deep layer of the total and lateral femorotibial compartment was found to be significantly greater than in bilateral KLG0 knees (Cohen's D 0.45–0.46), so that given specific conditions, the method appears to be sensitive to picking up difference between risk strata.

A strength of the current study was the use of a location-independent analysis method, which was previously shown to be more sensitive to differences in rates of change between different risk strata of knee OA than region-specific analysis [16]. Interestingly, participants with the greatest thinning scores (−1.5 ​mm and more) were found to exclusively be subjects from the “at risk” cohort, but none from the “non-exposed” cohort. The location-independent method has been successfully applied to unravel differences in cartilage thickness change between KLG0 knees with contralateral knee radiographic JSN compared with KLG0 knees without radiographic OA in the opposite knee [14]. The use of this type of method is particularly useful in studies of “preradiographic” or “early” OA, because at this stage cartilage loss may be more diffuse (or heterogeneous) and may involve the lateral compartment [14], whereas during established knee OA there often exists a predominance of the medial femorotibial compartment. Indeed, using this methodology, we were able to detect statistically significant difference in longitudinal cartilage thickness change between knees “at risk” vs. those “non-exposed” that did not become apparent when applying location-specific MRI or measures of radiographic JSW. The location-independent cartilage thinning score hence provides opportunity to study “structural progression” across the entire spectrum of the disease pathway, i.e. before, during and after incident ROA, with the advantage that prior to incident ROA collider bias does not apply when risk factors of progression are studied [9]. Please note that the definition of “pre-radiographic” made in this study was based on the 12-month follow-up radiographic classification made at the beginning of the observation interval. As some knees became KLG1 or developed definite incident radiographic knee OA during the study (≥KLG2), in some knees the change occurred concurrent with (rather than prior to) incident radiographic knee OA.

It appears puzzling that, despite the significant difference in cartilage thinning rates between knees “at risk” versus those “non-exposed”, the difference could not be attributed to commonly accepted risk factors that were used to select the “at risk” cohort, except for NRS pain. A potential explanation is that most of these demographic and clinical risk factors are not “strong” enough to explain the observed difference, and that there are other un-measured risk factors in play that determine the greater cartilage thickness change in KLG0 knees in the “at risk” cohort. This is supported by the observation that incident radiographic knee OA was only marginally greater over 48- (and even 96-month) follow-up in the “at risk” than in the “non-exposed” cohort. Another explanation may be that, despite the relatively long observation period of 3 years, this period may be too short in view of the relatively small rates of worsening observed in knees without radiographic OA at the beginning of the observation interval; identifying risk factors in KLG0 knees may hence require even longer observation intervals. In patients with established ROA of the knee, thinning scores were reported to amount to >1100 ​μm over only 2 years, and to >2000 ​μm when followed by total joint replacement [25]. This may also explain why pain frequency was found to be significantly associated with cartilage thinning over 1 year in knees with ROA (KLG2−4) [26], whereas it could not be identified as a risk factor of progression in the current study in KLG0 knees, and only NRS pain reached statistical significance when both cohorts were combined. Interestingly in this context, a study by Kraus et al. [27] suggested that composite trabecular bone texture (TBT) status, measured by fractal signature analysis in the medial subchondral tibia, was significantly associated with clinically relevant OA progression, i.e. a combination of symptom and radiographic worsening in knees with definite radiographic OA at baseline. In the same cohort, the 24 month changes of several biochemical liquid biomarkers predicted combined pain and radiographic progression status, the most predictive and parsimonious combinatorial model being one of time-integrated (baseline through 24-month change) concentrations of uCTXII, sHA and sNTXI. Baseline uCTXII and uCTXIα also were significantly predictive of combined progression case status [28].

Selecting KLG0 knees at risk of OA for disease-modifying osteoarthritis drug (DMOAD) therapy [29] remains challenging. The cartilage thinning score of the “at risk” cohort only exceeded that in the “non-exposed” reference cohort by 27% in the current study. We previously reported that KLG0 knees with contralateral radiographic JSN displayed a 71% greater thinning score that the “non-exposed” reference knees [14], which is a 35% greater thinning score than the “at risk” cohort studied here. These observations suggest that there exist subjects with at least one radiographically normal knee that display an increased “intrinsic susceptibility” to subsequent cartilage matrix loss. With the potential exception of NRS pain, however, the demographic and clinical variables studied here do not appear to be sufficiently related to future risk of progression to represent efficient selection criteria for clinical trials on preventive DMOADs.

In conclusion, in radiographically normal knees “at risk” of incident radiographic knee OA, the cartilage thinning score was observed to be significantly greater than that in a “non-exposed” reference cohort without risk factors. Superficial or deep cartilage T2 change scores, in contrast, did not differ significantly between both cohorts. Age, sex, body mass index, previous knee trauma and/or surgery, family history of joint replacement, presence of Heberden's nodes, repetitive knee bending were each not significantly associated with cartilage thinning, and only NRS pain reached statistical significance. This lack of association with a large set of common risk factors renders it challenging to select participants for testing a “preventive” DMOAD in a clinical trial based on demographic and clinical criteria alone. Further, the analysis of the effect of demographic and clinical risk factors on structural progression starting at the pre-radiographic phase, during which collider bias is irrelevant, requires long observation periods, despite the presence of sensitive measurement technology of structural progression.

## Author contributions

Study conception and design: FE, WW, AC, LS.

Acquisition of data: SM, WW, LS.

Analysis & interpretation of data: All authors.

Writing of first manuscript draft: FE.

Critical manuscript revision and approval of final manuscript: All authors.

WW had full access to all of the data in the study and takes responsibility for the integrity of the data and the accuracy of the data analysis.

## Role of the funding source

The cartilage thickness analysis was funded by an ancillary study to the OAI held by the Division of Rheumatology, 10.13039/100008250Feinberg School of Medicine, Northwestern University (R01 AR52918). The cartilage T2 analysis was funded by the Bundesministerium für Bildung und Forschung (BMBF – 01EC1408D (OVERLOAD-PREVOP)). The statistical analysis and writing of this article were independent from and not contingent upon approval from the study sponsors.

## Declaration of competing interest

Dr. Maschek and Dr. Wirth are part time employees and co-owners of Chondrometrics GmbH. Dr. Roemer was a part time employee of Chondrometrics during the funding period of the grant by the Bundesministerium für Bildung und Forschung (BMBF – 01EC1408D OVERLOAD-PREVOP) from 10/2015 until 02/2019 and is shareholder, CMO and Director of Research of Boston Imaging Core Lab (BICL), LLC. Dr. Duda and Dr. Sharma have no conflicts to declare. Dr. Eckstein is CEO/CMO and co-owner of Chondrometrics GmbH, and he has provided consulting services to Merck KGaA, Samumed, Tissuegene, Servier, Galapagos and Roche. He also has received speaker honoraria from Medtronic.
