# Supplementary material for: Which risk factors determine cartilage thickness and composition change in radiographically normal knees? – Data from the Osteoarthritis Initiative
Source: Osteoarthr Cartil Open. 2023 Apr 28;5(3):100365. doi: 10.1016/j.ocarto.2023.100365 (PMC10188628; doi:10.1016/j.ocarto.2023.100365)
Supplement: Multimedia component 1 [file mmc1.docx]

**Supplement Table 1: Baseline data in Kellgren Lawrence grade (KLG) 0 knees “at risk” of incident knee OA vs. “non-exposed” KLG0 reference knees for the subsample in which cartilage composition analysis (T2) was available**

**“At risk” (n = 59) “Non-exposed” (n = 52) Difference**

**Mean SD 95% CI N Mean SD 95% CI N P Cohen D**

Age (years) 65.1 8.6 62.9 67.4 59 54.6 6.7 52.8 56.5 52 <0.001 1.35

BMI (kg/m^2^) 27.3 4.1 26.2 28.4 59 24.6 3.3 23.7 25.5 52 <0.001 0.73

NRS (0-10) 1.6 2.1 1.0 2.1 59 0.2 0.5 0.0 0.3 52 <0.001 0.90

WOMAC Pain (0-20) 0.9 1.4 0.6 1.3 59 0.1 0.4 0.0 0.2 52 <0.001 0.76

ThC FTJ (mm) 7.3 1.2 7.0 7.6 59 7.2 1.1 6.9 7.5 52 0.552 0.11

ThC MFTC (mm) 3.5 0.6 3.3 3.6 59 3.4 0.5 3.2 3.5 52 0.316 0.19

ThC LFTC (mm) 3.9 0.6 3.7 4.0 59 3.9 0.6 3.7 4.0 52 0.840 0.04

T2 Deep (ms) 37.0 2.1 36.5 37.6 59 36.5 1.9 35.9 37.0 52 0.136 0.29

T2 Sf (ms) 47.4 2.8 46.7 48.2 59 45.4 2.4 44.7 46.1 52 <0.001 0.77

BMI = body mass index; NRS = numerica rating scale (0-10, with 0 being no pain, and 10 the worst pain), WOMAC = Western Ontario & Mc Master Universities Osteoarthritis Index pain component (0-20, with 0 being no pain, and 20 the worst pain); ThC = thickness of cartlage; FTJ = total femorotibial joint (ThC FTJ = ThC MFTC + ThC LFTC); MFTC = medial femorotibial compartment (ThC MFTC = ThC medial tibial + ThC medial weight-bearing femur; LFTC = ThC lateral tibial + ThC lateral weight-bearing femur; min JSW = minimum radiographic joint space width determined from fixed flexion radiographs; FL = fixed location: medial, X=0.225; lateral, X=0.750;
