# Supplementary material for: Which risk factors determine cartilage thickness and composition change in radiographically normal knees? – Data from the Osteoarthritis Initiative
Source: Osteoarthr Cartil Open. 2023 Apr 28;5(3):100365. doi: 10.1016/j.ocarto.2023.100365 (PMC10188628; doi:10.1016/j.ocarto.2023.100365)
Supplement: Multimedia component 2 [file mmc2.docx]

**Supplement Table 2: Three-year change in cartilage thickness in the 16 femorotibial subregions in Kellgren Lawrence grade (KLG) 0 knees “at risk” of incident knee OA vs. “non-exposed” KLG0 reference knees**

**“At risk” “Non-exposed” Difference**

**Mean SD 95% CI N Mean SD 95% CI N P Cohen D**

ThC cMT (µm) -17 112 -26 -9 678 -9 84 -28 10 77 0.524 -0.08

ThC eMT (µm) -13 86 -20 -7 678 -20 62 -34 -6 77 0.47 0.09

ThC iMT (µm) 3 112 -6 11 678 36 107 12 60 77 0.01 -0.30

ThC aMT (µm) -5 78 -11 1 678 -8 56 -20 5 77 0.80 0.03

ThC pMT (µm) -6 68 -11 -1 678 -11 64 -25 4 77 0.56 0.07

ThC ccMF (µm) 8 108 0 16 678 33 87 13 52 77 0.06 -0.23

ThC ecMF (µm) 32 90 25 39 678 23 67 8 38 77 0.42 0.10

ThC icMF (µm) -7 89 -13 0 678 14 62 0 28 77 0.046 -0.24

ThC cLT (µm) -41 144 -52 -30 678 -7 96 -29 15 77 0.044 -0.24

ThC eLT (µm) -12 92 -19 -5 678 2 78 -16 20 77 0.18 -0.16

ThC iLT(µm) -36 122 -45 -27 678 -38 130 -67 -8 77 0.93 0.01

ThC aLT (µm) -9 79 -15 -3 678 -12 84 -31 8 77 0.80 0.03

ThC pLT (µm) -32 84 -38 -25 678 -21 98 -43 1 77 0.31 -0.12

ThC ccLF (µm) 14 101 6 21 678 27 71 11 43 77 0.28 -0.13

ThC ecLF (µm) 11 96 4 18 678 16 93 -5 38 77 0.64 -0.06

ThC icLF (µm) 5 89 -2 12 678 10 81 -9 28 77 0.67 -0.05

ThC = thickness of cartilage; c = central; e = external; I = internal; a = anterior; p = posterior; MT = medial tibia; cMF = weight-bearing medial femur;
LT = lateral tibia; cLF = weight-bearing lateral femur
